# Viral Infection in Adults with Severe Acute Respiratory Infection in Colombia

**DOI:** 10.1371/journal.pone.0143152

**Published:** 2015-11-17

**Authors:** Yuly Andrea Remolina, María Mercedes Ulloa, Hernán Vargas, Liliana Díaz, Sandra Liliana Gómez, Alfredo Saavedra, Edgar Sánchez, Jorge Alberto Cortés

**Affiliations:** 1 Department of Internal Medicine, Faculty of Medicine, National University of Colombia, Bogotá, Colombia; 2 Public Health Laboratory, District Health Department, Bogota, Colombia; 3 Infectious Disease Research Group, Faculty of Medicine, National University of Colombia, Bogotá, Colombia; Kliniken der Stadt Köln gGmbH, GERMANY

## Abstract

**Objectives:**

To identify the viral aetiology in adult patients with severe acute respiratory infection (SARI) admitted to sentinel surveillance institutions in Bogotá in 2012.

**Design:**

A cross-sectional study was conducted in which microarray molecular techniques for viral identification were used on nasopharyngeal samples of adult patients submitted to the surveillance system, and further descriptions of clinical features and relevant clinical outcomes, such as mortality, need for critical care, use of mechanical ventilation and hospital stay, were obtained.

**Setting:**

Respiratory infections requiring hospital admission in surveillance centres in Bogotá, Colombia.

**Participants:**

Ninety-one adult patients with acute respiratory infection (55% were female).

**Measurements:**

Viral identification, intensive care unit admission, hospital stay, and mortality.

**Results:**

Viral identification was achieved for 63 patients (69.2%). Comorbidity was frequently identified and mainly involved chronic pulmonary disease or pregnancy. Influenza, Bocavirus and Adenovirus were identified in 30.8%, 28.6% and 18.7% of the cases, respectively. Admission to the intensive care unit occurred in 42.9% of the cases, while mechanical ventilation was required for 36.3%. The average hospital stay was 9.9 days, and mortality was 15.4%. Antibiotics were empirically used in 90.1% of patients.

**Conclusions:**

The prevalence of viral aetiology of SARI in this study was high, with adverse clinical outcomes, intensive care requirements and high mortality.

## Introduction

Acute respiratory infection is one of the main causes of hospitalization and death worldwide, although identification of the aetiological agent is not achieved in a majority of cases. Instead, the infections are treated empirically and often successfully with antimicrobial therapy. Nonetheless, the roles of viruses in the aetiology of these infections are becoming clear, especially after the 2009 pandemic of the new Influenza A subtype H1N1 [1]. The presence of a virus does not imply either a more benign clinical course or that systemic inflammatory responses or complications will be absent [2].

Due to its implications for Public Health, the efforts in reinforcing and improving the epidemiological surveillance of respiratory infections have increased. Under this initiative, countries have developed surveillance systems by following cases of influenza-like illness and severe acute respiratory infections (SARIs), which are clinically diagnosed among patients with fever, coughing or sore throat, difficulty breathing and the need for hospitalization [3]. The main aims of surveillance have been to provide information on circulating viruses and the susceptibility of Influenza to available antivirals and also to promote and define vaccination needs in different populations.

The true impact of viral infections in the aetiology of acute respiratory disease requiring hospitalization is unknown [4]. The aims of this study were to identify viral aetiologies in hospitalized adult patients with SARI in Bogotá in 2012 and to describe the characteristics and clinical outcomes among these patients.

## Materials and Methods

### Study scenario

This study was performed in Colombia’s capital city of Bogotá, which is located in the Andes in South America, near the equator and 2,600 metres above sea level. A total of 7 tertiary care hospitals performing sentinel surveillance of SARI during 2012 participated in the study. Such hospitals forwarded all respiratory samples from patients with SARI to the District Health Department. SARI was defined as any respiratory infection with a possible viral and/or bacterial origin requiring inpatient management and a clinical presentation of fever of less than 14 days after onset and higher than 38°C, shortness of breath, cough, hypoxia and systemic compromise (systemic inflammatory response syndrome or organ failure), depending on symptom severity [5]. The samples were taken via nasopharyngeal aspiration or swab. The samples were sent, together with the required basic epidemiological data collection form, through the Epidemiological Surveillance System (Sistema de Vigilancia Epidemiológica Nacional—Sivigila). Samples were sent in a viral transport medium to the Public Health Laboratory of the District Health Department, where they were stored between 4 and 8°C in refrigerators intended for this purpose.

### Study design

This descriptive cross-sectional study was performed in Bogotá in 2012 to determine the prevalence of viruses in adult patients with SARI of less than 15 days of evolution using a microarray technique. The study also describes clinical characteristics and outcomes such as mortality, the need for intensive care, the use of mechanical ventilation and the length of hospital stay. Inclusion criteria consisted of patients over 18 years of age who provided a respiratory sample in 2012 at one of the hospitals performing sentinel surveillance. Patients with incomplete medical records from the sampling institution were excluded. The following research and ethics committees of each participating institution approved the study: Comité de Investigaciones en Salud (Hospital de Occidente Kennedy, III Nivel), Comité de Ética de Investigación (Hospital El Tunal ESE), Comité de Ética en Investigación (Hospital Santa Clara Empresa Social del Estado III Nivel), Comité de Investigaciones y Ética (Hospital Universitario San Ignacio), Comité de Ética de la Investigación (Hospital Universitario Clínica San Rafael) and Comité de Ética en Investigación Clínica (Fundación Cardioinfantil, Instituto de Cardiología). One institution does not have a formal Research and Ethics Committee but instead has an office in charge of the administrative procedures for research (Oficina de Educación, Hospital de Suba ESE II Nivel). Because this was a retrospective study that involved minimal risk, a waiver of informed written and oral consent was sought from each research and ethics committee. This waiver was granted by each of the ethics and research committees (Hospital de Occidente Kennedy, III Nivel; Hospital El Tunal ESE; Hospital Santa Clara Empresa Social del Estado III Nivel; Hospital Universitario San Ignacio; Hospital Universitario Clínica San Rafael; and Hospital de Suba ESE, II Nivel), with the exception of one institution that requested written informed consent to access medical records (Fundación Cardioinfantil, Instituto de Cardiología). Written consent was obtained from 25 participants or their proxies (in case of death) from that institution (39 patients were not found, did not respond or refused to provide written consent). Written informed consent, in the cases in which was requested and obtained, was kept at Universidad Nacional de Colombia.

### Sample selection

A total of 288 nasopharyngeal aspiration or swab samples were taken during 2012 from adult patients of the 7 hospitals. The sample was randomly selected, with the only inclusion criteria being patients older than 18 years of age with SARI reported to the surveillance system during that year. A sample size calculation found that 117 respiratory samples were needed for a prevalence of 25%, with the poorest accepted result of 20%, 90% power and a 95% confidence level. However, because 150 molecular tests could be processed, the sample was increased to 150 patients. Subsequently, the medical records were reviewed, and after the approval and authorisation of the research and ethics committee of each institution, the information was gathered from each hospital.

### Microarray processing

A microarray diagnostic assay using CLART^®^ PneumoVir equipment by Genomica (Madrid, Spain) was used to identify the viruses involved [6]. This assay detects and characterises viruses that most frequently cause respiratory symptoms in humans. The following viruses were analysed: Respiratory Syncytial Virus (RSV) A and B, Influenza A (H1N1, H3N2, 2009 A/H1N1 pdm), B and C virus, Parainfluenza virus subtypes 1, 2, 3, 4A and 4B, Metapneumovirus A and B, Adenovirus, Enterovirus, Rhinovirus, Coronavirus subtype 229E and Bocavirus. This kit amplifies specific fragments of the viral genome via a reverse transcription polymerase chain reaction (PCR) or via a PCR with hybridisation detection using specific capture probes [7].

### Variables and definitions

The following variables were collected from the patients’ medical histories according to international definitions: age, gender and comorbidities such as chronic obstructive pulmonary disease (COPD), diabetes mellitus and heart failure. The inclusion criteria took into account the systemic inflammatory response syndrome (SIRS), which was defined as a heart rate over 90 beats per minute, a breathing rate of over 20 breaths per minute, leukocytosis of over 12,000 cells per millilitre or leukopenia of fewer than 4,000 cells per millilitre and fever; pneumonia among SARI patients was diagnosed from radiological findings of consolidation or alveolar infiltrates. The Confusion, Urea, Respiratory Rate and Blood Pressure (CURB)-65 scores for pneumonia were applied using the criteria of age greater than or equal to 65 years, impaired consciousness, blood urea nitrogen (BUN) over 20 mg/dl, breathing rate above 30 breaths per minute and a systolic arterial pressure under 90 mmHg or a diastolic pressure under 60 mmHg [8]. Additionally, severe pneumonia was considered among patients complying with the following major and minor criteria of the American Thoracic Society/Infectious Disease Society of America (ATS/IDSA): shock, need for mechanical ventilation, thrombocytopenia (platelets under 100,000), an Arterial Oxygen Partial Pressure to Fractional Inspired Oxygen Ratio (PaO_2_/FiO_2_) ratio, multilobar compromise, impaired consciousness, leukopenia (under 4,000) and BUN over 20 mg/dl [9]. Shock was also considered when the systolic arterial pressure was below 90 mmHg or when the diastolic arterial pressure was under 60 mmHg. Moderate oxygenation impairment was defined by a PaO_2_/FiO_2_ below 220 mmHg and over 160 mmHg, and severe oxygenation impairment was defined by an index less than or equal to 160 mmHg. Institutions that identified viral antigens following institutional protocols performed and interpreted these tests in their hospitals. Colonisation of the airway was defined as the presence of microorganisms in a Gram test or in a culture of the airway. Gram tests and sputum cultures or other respiratory samples were performed based on clinical decisions made by the doctor from the institution. A viral co-infection diagnosis was made when 2 or more different viruses were identified in the same sample.

### Statistical analysis

The chi-square test and Fisher’s exact test were used to compare categorical variables, and the comparison of continuous variables was performed with either Student’s *T* test or the Mann-Whitney *U* test on a case-by-case basis. The variable analysis was performed using Stata (ver. 11.0), and *P* values less than 0.05 were considered significant. Odds ratios (OR) with corresponding 95% confidence intervals (CI) were used to analyse outcomes.

## Results

**Fig 1:**
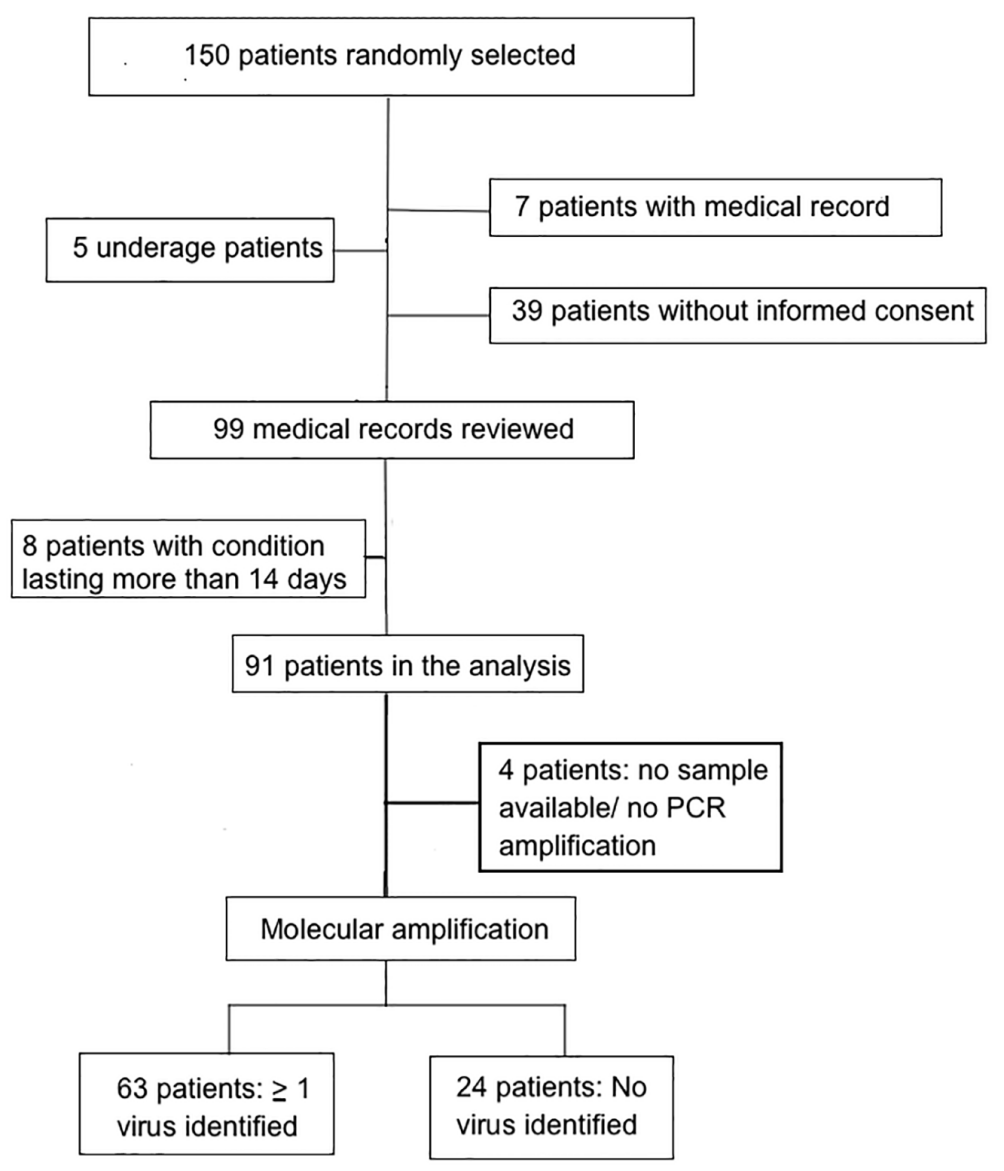
Flow diagram of subject identification, sample processing and categorisation. Four patients did not have molecular results (right arm).

In 2012 in Bogotá, 288 samples from respiratory secretions were collected in the sentinel surveillance system from patients over 18 years of age. Through randomised sampling, 150 cases of SARI were selected from those respiratory samples (Fig 1). The medical histories of 99 patients with SARI reported through the surveillance system were examined. These histories were obtained from 7 Bogota hospitals in 2012 according to the following distribution: 45 (45.5%) were from the San Rafael University Hospital Clinic, 23 (23%) were from Fundación CardioInfantil, 10 (10%) were from Suba Hospital, 9 (9.1%) were from El Tunal Hospital, 6 (6%) were from Santa Clara Hospital, 3 (3%) were from San Ignacio University Hospital, and 3 (3%) were from the Occidente de Kennedy Hospital. Of the 99 patients, 8 showed disease progression beyond 14 days and were dismissed from the final analysis (Fig 1).

Of the 91 patients included in the analysis, 50 (55%) were female; the patients’ average age was 50.6 years (range, 18 to 95 years). Table 1 shows the most frequent comorbidities identified. The average length of disease progression was 5.1 days, with a minimum duration of hours and a maximum duration of 14 days (Table 2). Radiographical abnormalities were observed in 65 patients. The following radiological findings of chest images were described in the medical histories, in order of frequency: 32 patients had interstitial infiltrates (35.1%), 27 had alveolar infiltrates (29.6%), 26 had consolidation (28.6%), 21 had multilobar compromise (23.1%), and 19 had pleural effusion at admission (20.9%). Pleural effusion was found in 15.3% of the patients with alveolar infiltrates and 13.2% of the patients with interstitial infiltrates. We had no information regarding the performance of CT scans among the patients. SIRS was diagnosed in 66 (72.5%) patients. Pneumonia was detected in 38 patients (41.7%), of whom 15 required mechanical ventilation. Regarding the CURB-65 score, a score of 0 was observed in 13 patients (34.2% of patients with pneumonia), a score of 1 was observed in 15 patients (39.5%), a score of 2 was observed in 7 patients (18.4%) and a score of 3 was observed in 3 patients (7.9%). Shock at admission was present in 11 (12.1%) patients. Hyponatraemia was detected in 21 (23.1%) patients, 11 (12.1%) patients presented moderate oxygenation impairment, and 20 (22%) patients presented severe oxygenation impairment.

**Table 1:** Characteristics of patients included in the study.

Variable	Number of patients	% of patients
Average age (years)	91	50.6%
Female gender	50	55.0%
Community-acquired infection	90	98.9%
COPD	22	24.2%
Previous hospitalization	14	15.4%
Smoking	13	14.3%
Pregnancy	11	12.1%
Diabetes mellitus	9	9.9%
Heart failure	9	9.9%
Immunosuppression	5	5.5%
Chronic kidney disease	4	4.4%
Stroke	4	4.4%
Chronic liver disease	3	3.3%
Cancer	3	3.3%
Alcoholism	2	2.2%

**Table 2:** Comparative clinical characteristics of the patients, by group.

Variable	Non-virus patients % (n)	Virus patients % (n)
Number of patients	24	63
Progression time (average in days)	5.1	5.3
Cough	75.0% (18)	87.3% (55)
Fever	70.8% (17)	71.4% (45)
Expectorate	62.5% (15)	52.4% (33)
Wheezing	25.0% (6)	39.7% (25)
Previous antibiotic treatment	29.2% (7)	17.5% (11)
Impaired consciousness	8.7% (2)	6.5% (4)
HR (beats/min)	96.3	98.6
BR (breaths/min)	20.9	20.6
SAP (mmHg)	122.7	118.0
DAP (mmHg)	72.6	71.1
Temperature (°C)	37.2	36.7
Neutropenia during hospitalization	4.2% (1)	5.0% (3)
SIRS ≥2	66.7% (16)	74.6% (47)
Pneumonia	33.3% (8)	44.4% (28)
Severe pneumonia	62.5% (15)	60.1% (38)
Shock	8.7% (2)	12.7% (8)

Among the SARI patients, 82 (90.1%) were treated with antibiotics; patients received between 1 and 7 antibiotics, with an average of 2.3 antibiotics per patient. Antibiotics were started a median of 1 day after admission (range, 0 to 21 days, 95% CI: 0–7 days). The beta-lactam group was the most frequently used. Oseltamivir was used in 72.5% of cases (66 patients), with a minimum duration of 1 day, a maximum duration of 10 days and an average duration of 4.2 days. Oseltamivir was started a median of 1 day after admission (range, 0 to 21 days, 95% CI: 0–9 days).

### Viral identification

At least one virus was identified in 63 patients (Table 3). Influenza virus was the most common and was isolated in 28 patients (30.8%), of whom 21 (75%) had Influenza A and 7 (25%) had Influenza B (Table 3). Of the Parainfluenza virus subtypes identified, five cases were PIV 3, and one case was PIV 1.

**Table 3:** Identified viruses.

Isolated virus	N of patients	% of patients
Virus	63	69.2%
Influenza	28	30.8%
Bocavirus	26	28.6%
Influenza A	21	23.1%
Adenovirus	17	18.7%
Influenza A 2009/ H1N1 pdm	7	7.7%
Influenza B	7	7.7%
Metapneumovirus	6	6.6%
Parainfluenza	6	6.6%
Respiratory syncytial virus	5	5.5%
Rhinovirus	5	5.5%
Influenza A subtype H2N3	2	2.2%
Coronavirus	2	2.2%
Mixed isolate (more than 1 virus)	26	41.3%

In the group with more than one viral infection (26 patients), 7 patients had a co-infection of 3 viruses in 7 different combinations: 1 patient had Bocavirus, Coronavirus and Influenza; 1 had Bocavirus, Influenza and Adenovirus; 1 had Bocavirus, Influenza and Rhinovirus; 1 had Adenovirus, Metapneumovirus and Rhinovirus; 1 had Bocavirus, Influenza and Parainfluenza (PIV 3); 1 had Adenovirus, Parainfluenza (PIV 3) and RSV; and 1 had Adenovirus, Influenza and Parainfluenza (PIV 1). There were 19 patients infected with 2 viruses, with co-infection of Bocavirus and Influenza in 6 patients being the most frequent; 2 patients were co-infected with Influenza and RSV, 2 with Adenovirus and Bocavirus, 2 with Adenovirus and Influenza, 2 with Bocavirus and Metapneumovirus, 1 with Bocavirus and RSV, 1 with Influenza and Metapneumovirus, 1 with Adenovirus and Metapneumovirus, 1 with Adenovirus and Rhinovirus, and 1 with Parainfluenza (PIV 3) and Rhinovirus.

### Viral seasonality

**Fig 2:**
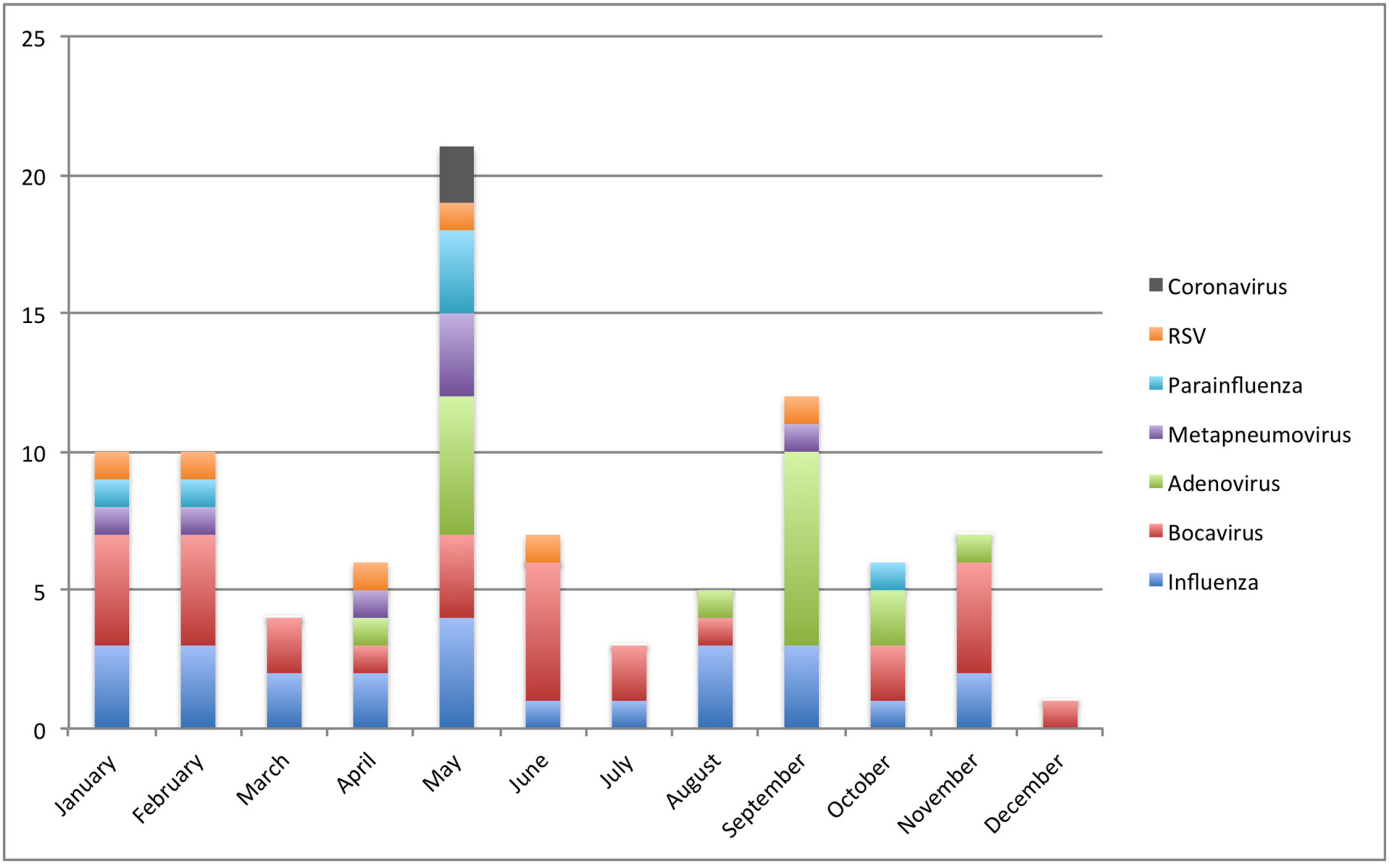
Monthly frequency of patients with viral Identification through 2012.

Influenza and Bocavirus were identified throughout the year, though cases of Influenza were commonly found in May, August and September, and cases of Bocavirus were commonly found in June and November. Influenza B was identified only in the second semester, while the two cases of Influenza A/H2N3 were seen in May. Adenovirus was seen only during the rainy season (April to May and August to November); most cases of Metapneumovirus, Parainfluenza and RSV were seen only in the first semester, specifically in April and May; the two cases of Coronavirus were identified in May (Fig 2).

### Comparison between patients with and without viral identification

Patients with and without viral detection did not differ in terms of comorbidity, with the exception of the frequency of diabetes mellitus. Diabetes was detected in 4.8% of patients with viral detection and 20.8% of patients without viral detection (Fisher’s exact test, *P* = 0.034).

Of the patients with pneumonia, a virus was identified in 28 patients (73.7%). Severe pneumonia was diagnosed in 62.5% of patients without viral detection and 60.1% of patients with viral detection. No significant differences were observed between the groups with and without viral detection with regards to SIRS, neutropenia during hospitalization or shock.

Of the 22 patients with SARI and a history of COPD, viruses were identified in 16 (72.7%), 5 of whom had viral co-infections. The viruses were distributed as follows: 6 cases of Bocavirus, 4 of Adenovirus, 3 of Influenza A, 3 of Parainfluenza, 3 of RSV, 2 of Metapneumovirus, 1 of Coronavirus and 1 of Rhinovirus. Of the 91 patients, 13 were active smokers and 9 (69.2%) had a viral infection, with Bocavirus being the most frequently isolated (33.3%) virus.

### Additional microbiological findings

During hospitalization, 33 patients (36.3%) had blood cultures taken. There were 3 positive cases (10% of the blood cultures); these consisted of two cases of bacteraemia by *Streptococcus* of the *viridans* group and one case by *Staphylococcus aureus*. Sputum Gram stains were performed in 22 patients (24%), and these showed diverse flora of Gram-positive cocci and bacilli or Gram-negative coccobacilli in 15 cases (68% of those with positive Gram stains). Positive sputum cultures were observed in 13 patients, with the following viruses identified: *Haemophilus influenzae* in 7 cases; *Streptococcus pneumoniae*, *Moraxella catarrhalis*, *H*. *parainfluenzae*, *Klebsiella pneumoniae* in two cases each; and *S*. *aureus*, *Bordetella pertussis*, *Stenotrophomonas maltophilia* in one case each. More than one microorganism was identified in 4 cases. Positive Gram staining was more frequent among patients with COPD (41% vs. 18.8%, *P* = 0.03), and colonisation of the airways was also more frequent in these patients (31.8% colonisation vs. 18.8%, Fisher’s exact test, *P*<0.03). Bacterial colonisation was identified together with viral identification in 14 patients (15.3%).

### Outcomes

With regards to complications, pleural effusion developed in 12.5% of patients without viral identification and 19% of patients with viral identification; closed thoracotomy was required in 4.2% of patients without viral identification and 3.2% of patients with viral identification; inpatient infection was present in 8.3% and 3.2% of patients without and with viral identification, respectively; and underlying disease complications were found in 25% of patients without viral identification and 38.1% of patients with viral identification. These differences were not statistically significant. Patients with chronic pneumopathy showed more complications in their underlying pathologies (77.3% vs. 20.3%, OR: 13.5; CI 95%: 3.5–52.7).

A total of 14 (15.4%) patients died. However, two cases included either an inadequate sample for PCR or no PCR amplification; thus, the outcome analysis was performed for 12 patients. Mortality was reported for 4 cases (16.7%) in the group without viral identification and 8 cases (12.7%) in the group with viral identification (*P* = 0.23, OR: 0.72; 95% CI: 0.17–3.68) (Table 4). Mechanical ventilation was required for 33 patients (36.3%); this intervention was invasive in 24 (26.4%) patients, non-invasive in 13 (14.3%) patients and invasive and non-invasive in 4 (3.6%) patients. This outcome occurred among 7 patients (29.2%) without viral identification and 23 patients (36.5%) with viral identification (*P* = 0.41, OR: 1.4; 95% CI: 0.46–4.59). The average length of mechanical ventilation was 7 days, with a minimum of 1 day and a maximum of 20 days. Thirty-nine (42.9%) patients were admitted to the intensive care unit (ICU); 3 had unsuitable samples or non-amplified PCR results, 9 (37.5%) were from the group without an isolated virus, and 27 (42.9%) were from the group with an isolated virus (*P* = 0.21, OR 1.25; 95% CI: 0.43–3.75). One case with viral identification was readmitted to the unit with an identification of Influenza subtype AH1N1. The average length of ICU stay was 8.4 days, with a range between 1 and 21 days. The group with an isolated virus had an average ICU stay of 3.8 days, with a range between 1 and 21 days, and the group without an isolated virus had an average stay of 2.8 days, with a range between 1 and 13 days (*P* > 0.05). The average hospital stay in the SARI group was 9.9 days, with a range between 1 and 45 days, with average stays of 10.6 and 9.8 days for patients with and without a virus, respectively (*P* = 0.6). Among patients without a virus, the hospital stay varied between 1 and 45 days, while patients with a virus had hospital stays that varied between 1 and 28 days.

**Table 4:** Outcomes of included patients.

Outcome	% of non-virus patients	% of virus patients	OR	Confidence interval
Death	16.7%	12.7%	0.72	0.17–3.68
Mechanical ventilation	29.2%	36.5%	1.4	0.46–4.59
Admission to ICU	37.5%	42.9%	1.25	0.43–3.75
Hospital stay (average days)	10.6	9.8	-	-

Of the 8 patients from the viral infection group who died, Bocavirus was isolated in 5, Influenza was isolated in 4 (all cases of Influenza A), Metapneumovirus was isolated in 3, and RSV was isolated in 1. Of the patients with Bocavirus isolation, 19.2% died. Death occurred in 14.3% of those in whom Influenza was identified, 50% of those in whom Metapneumovirus was identified and 20% of those in whom RSV was identified. Among patients with viral identification who died, 5 (62.5%) had viral infections of 2 or more viruses (*P* = 1.7, OR: 2.4; 95% CI: 0.46–18.89), and fewer had only one virus detected. No statistically significant differences were observed in the outcomes of the remaining patients with mixed viral infection, which does not confirm higher morbidity in patients with more viruses isolated.

## Discussion

The diagnosis of acute respiratory infections is common and apparently easy to perform; however, the determination of the infection’s causal agent is more complex, as current diagnostic tools are limited and rarely available in primary health care centres or even in hospitals in much of the world [10]. The role of viruses and their prevalence is a matter of debate and discussion, and findings vary worldwide. The results from studies in New Zealand, Spain and more recently in the United Kingdom report prevalence rates of 28% [11], 18% [12],and 44% [13], respectively. In our study, viruses were identified as the most frequent causal agents of SARI requiring hospitalization in 2012, with most cases showing a high rate of viral co-infection, a high degree of morbidity, prolonged hospital stays and frequent needs for ICU management and mechanical ventilation.

The definition of SARI used in this study is of great utility from an epidemiological surveillance perspective, which is known as syndrome surveillance or surveillance of syndromes [14]. Nonetheless, the results reported here demonstrate its clinical relevance and potential utility in this field, as it appears to identify potentially severe patients and those with high complication rates; thus, the definition’s routine use should be considered during the performance of clinical duties. Furthermore, current challenges in the epidemiological surveillance of viral respiratory tract infections include the early and fast identification of aetiological agents, especially at the beginnings of outbreaks, and the optimal and timely management of a large number of samples [14]. Our study suggests that molecular technology makes it possible to closely follow circulating viruses in these groups of patients. In contrast to the epidemiological definition used, the CURB-65 index of pneumonia was not useful, as only 10 (26%) scored for general hospitalization and none scored for ICUs using these criteria. This finding contrasts with the high number of our patients who required ICU care, which agrees with findings in the literature that applied this scale in cases of viral pneumonia during the Influenza A subtype H1N1 pandemic of 2009; these results demonstrate its low value for detecting either severity or the need for ICU admission in patients with viral pneumonia [15]. These findings represent a marked difference in severity stratification between Influenza-related pneumonia and pneumonia caused by other aetiological agents, indicating the importance of clinical judgement in this scenario [16]. The application of these scales in non-Influenza viral pneumonia has yet to be assessed. Moreover, a recent multicentre study of adults with radiographically confirmed pneumonia has shown that viruses are now the most commonly identified pathogens. Human Rhinovirus and Influenza virus are more frequently found than *Streptococcus pneumoniae* [17]. Together, viruses represent a quarter of patients and more than half of the pathogens identified.

The mortality rate was relatively high for patients both with and without community-acquired pneumonia. A study performed in the United States reported a low mortality rate in patients hospitalized for viral infection; nonetheless, bacterial co-infection increased both the morbidity and the mortality [18]. This variable should be considered for our patients. Countries with marked seasons report a correlation of Influenza with high mortality due to respiratory infections, which is usually related to community-acquired pneumonia [19]. This seasonal pattern of acute respiratory infections, especially viral infections due to Influenza, is dependent on temperature, humidity and host factors, such as serum vitamin D levels [20]. Colombia is located in the tropics and thus lacks seasons; however, this study confirms that these infections, especially those caused by Influenza and RSV infections, increase during the rainy season in countries at these latitudes [21]. This pattern shows the importance of epidemiological surveillance, especially during the seasons when viruses circulate, because it may increase control and prevention strategies such as timely vaccinations.

Techniques based on identifying nucleic acids, such as those used in this study, can obtain more rapid and precise results for diagnosing viral infections in order to provide appropriate and managed medical care [22]. There is limited information on the diagnostic utility of these new tests. A study by Sultankulova et al. compared viral isolation of Influenza A with DNA microarray technology, reverse transcription PCR and real-time PCR [23]. Microarray technology showed a higher sensitivity (99.5%) and similar specificity (98.5%) to real-time PCR. However, another study with the same microarray kit used in this study showed a high specificity (100%) and low sensitivity (52%) in the clinical scenario of atypical pneumonia [24]. A study in Japan using near patient automated microarray technology showed not only a higher sensitivity and specificity compared to immunochromatographic antigen detection (the gold standard used was virus isolation) but also quicker results for children infected with Influenza and RSV [25]. A recent multicentre study in the United States using real-time PCR technology was able to precisely and reproducibly detect all Adenovirus infections in a group of children and adults with respiratory infections, which increased the possibility of establishing a clear diagnosis [26]. Taken together, use of molecular technology for the diagnosis of viral infections can improve detection and identify the cases in which antibiotic use might be inadequate. The use of antibiotics in acute respiratory infections is indiscriminate and excessive, and according to worldwide literature, is employed in more than half of all respiratory infections [27]. This study determined that 90% of the cases are treated with antibiotics, although most of the infections were viral in origin. Additional strategies, such as the measurement of procalcitonin, can identify patients who would not benefit from antibiotic therapy [28].

This study has several limitations. Although it was multicentre in design, the information was gathered from only one city in Colombia. One important limitation is that it is a retrospective study; thus, it was not possible to control viral and bacterial sampling, and there was limited access to relevant data, such as previous Influenza or pneumococcal vaccination history or co-morbidity measurement. The sample size was smaller than expected, although the prevalence of viral infection was higher than expected. Together, all of these limitations lead to difficulties in establishing significant comparisons among the groups and in appropriately assessing the impact of co-morbidity and bacterial co-infection in the outcomes, especially in relation to the role of pneumococcus [29]. Other limitations include the difficulty of defining the actual roles of certain viruses, such as Bocavirus and Rhinovirus, in respiratory infection, which are also detected in asymptomatic patients according to descriptions in the literature [4]. The empirical use of antibiotics in most cases, without using additional tools to confirm bacterial infection, is also a limitation.
